# Serial Presepsin Measurement as a Predictor of In-Hospital Mortality in Older Adults with Hip Fractures

**DOI:** 10.3390/life16081316

**Published:** 2026-08-12

**Authors:** Bünyamin Arı, Fatmagül Can, Umur Batak, Turan Cihan Dülgeroğlu

**Affiliations:** 1 Department of Orthopedics and Traumatology, Faculty of Medicine, Kütahya Health Sciences University, Kütahya 43000, Türkiye; 2 Department of Medical Biochemistry, Faculty of Medicine, Kütahya Health Sciences University, Kütahya 43000, Türkiye

**Keywords:** presepsin, hip fracture, older adults, in-hospital mortality, biomarker, prognosis, innate immune activation

## Abstract

Serial presepsin measurements were evaluated for their prognostic value compared with conventional laboratory markers in predicting in-hospital mortality among older adults with hip fractures. This prospective observational study included 85 patients aged ≥65 years admitted with acute hip fractures between December 2025 and May 2026. Residual serum remaining after routine clinical laboratory sampling was obtained on admission (Day 1), Day 3, and Day 5; serum presepsin was measured by commercial ELISA, and routine parameters (C-reactive protein [CRP], white blood cell count, lymphocytes, monocytes, platelets, and liver enzymes) were analysed in the hospital laboratory. Renal function was assessed by serial creatinine and estimated glomerular filtration rate (eGFR). Patients were classified as survivors or non-survivors according to in-hospital outcome. Temporal trajectories were modelled with a linear mixed-effects model fitted to log-transformed presepsin, receiver operating characteristic (ROC) analysis assessed predictive performance, and internal validity was examined by bootstrap resampling. Of the 85 patients, 68 survived and 17 died during hospitalization; all deaths occurred between hospital days 5 and 12. Presepsin diverged progressively between groups, reaching significantly higher concentrations in non-survivors by Day 5 (median 207.70 vs. 147.21 ng/L; *p* < 0.001), with a Day 5 group-by-time interaction ratio of 1.76 (95% CI 1.38–2.25; *p* < 0.001). Day 5 presepsin showed the highest predictive accuracy (AUC = 0.868, 95% CI 0.774–0.945; optimism-corrected AUC 0.866), significantly outperforming CRP (AUC = 0.606; *p* = 0.003) and platelet count (AUC = 0.485; *p* < 0.001). The association persisted after adjustment for age, ASA class, fracture type, and eGFR, and Day 5 presepsin added discrimination to a baseline clinical model (ΔAUC 0.125; *p* = 0.015). The Youden-derived cutoff of 169.54 ng/L was unstable across bootstrap resamples (95% range 169.5–207.7 ng/L). Serial presepsin measurement, particularly on Day 5, is associated with in-hospital mortality in this population; these exploratory findings require external validation before any clinical application can be considered.

## 1. Introduction

Hip fractures are a major public health problem in the older adult population and are associated with high rates of morbidity, mortality, and healthcare costs worldwide [1,2]. Despite advances in surgical techniques, anesthesia, and perioperative care, in-hospital and early mortality following hip fractures remain substantial, particularly in patients aged 65 years and older [3]. Early identification of patients at increased risk of adverse outcomes is therefore crucial to optimize perioperative management and improve survival [4].

Mortality after hip fracture is influenced not only by chronological age and comorbidities but also by the magnitude and persistence of the systemic inflammatory response triggered by trauma and surgical stress [5,6]. Routine laboratory parameters such as white blood cell count, platelet count, lymphocyte count, and C-reactive protein (CRP) are commonly used to evaluate inflammatory and immune responses in clinical practice. Persistent lymphopenia, impaired platelet recovery, and sustained elevation of CRP have been associated with poor prognosis in older adults with hip fractures [7]. However, these conventional markers often lack sufficient sensitivity and specificity to reliably identify high-risk patients at an early stage, highlighting the need for additional prognostic biomarkers [8].

Given the limitations of conventional inflammatory markers, increasing attention has been directed toward biomarkers that more directly reflect innate immune activation.

Presepsin, a soluble subtype of cluster of differentiation 14 (CD14), is released during activation of the innate immune system and has emerged as a promising biomarker for assessing disease severity and mortality in sepsis and systemic inflammatory conditions [9,10]. Due to its rapid kinetics and close association with monocyte–macrophage activation, presepsin may reflect systemic inflammatory burden more accurately than traditional laboratory markers [11]. Although the prognostic value of presepsin has been well established in infectious diseases, its role in non-infectious conditions such as orthopedic trauma has not been adequately investigated. This is particularly true of older adults with hip fractures, a population in which management strategies and outcomes vary considerably across fracture patterns and treatment options [12] and in which serial biomarker data remain scarce [13,14].

Therefore, the aim of the present study was to evaluate the prognostic significance of serial presepsin measurements in comparison with routine laboratory parameters for predicting in-hospital mortality in older adults admitted with hip fractures [15]. We hypothesized that presepsin, especially when measured serially during the early hospital course, would outperform conventional inflammatory markers in identifying patients at increased risk of in-hospital death.

## 2. Materials and Methods

### 2.1. Study Design and Population

This prospective observational study included patients aged 65 years and older who were admitted to our institution with acute hip fractures between December 2025 and May 2026. Enrolment commenced only after institutional ethics committee approval had been obtained. Hip fractures were classified as cervical, basicervical, intertrochanteric, or subtrochanteric on the basis of radiological findings and operative reports. No additional venipuncture was performed for study purposes; presepsin was measured exclusively on residual serum remaining after routine clinical laboratory testing. Patients were followed throughout their hospital stay and categorized into two groups according to in-hospital outcome: survivors (discharged alive) and non-survivors (death during hospitalization).

### 2.2. Inclusion and Exclusion Criteria

**Figure 1:**
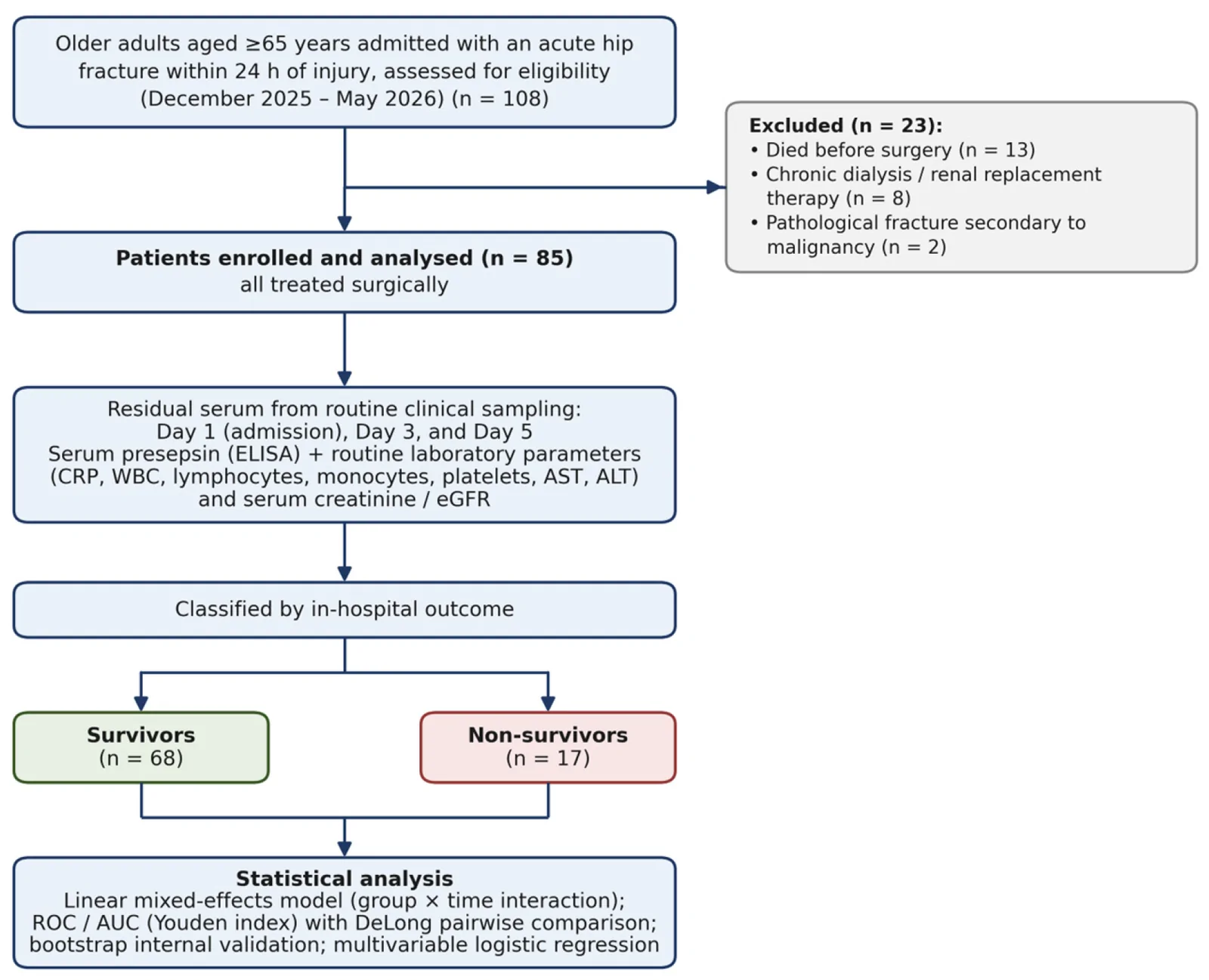
Study flowchart. Of 108 older adults aged ≥65 years admitted with an acute hip fracture within 24 h of injury and assessed for eligibility, 23 were excluded (13 died before surgery, 8 were receiving chronic dialysis, 2 had a pathological fracture secondary to malignancy) and 85 were enrolled. Residual serum for presepsin (ELISA) and routine laboratory parameters (CRP, WBC, lymphocytes, monocytes, platelets, AST, ALT) together with serum creatinine were obtained serially on days 1, 3, and 5 of hospitalization. Patients were classified according to in-hospital outcome as survivors (*n* = 68) or non-survivors (*n* = 17). Predictive performance was assessed by ROC/AUC analysis with DeLong pairwise comparison, bootstrap internal validation, and multivariable logistic regression.

Patients aged ≥65 years with an acute hip fracture who were admitted within 24 h of injury were eligible for inclusion. Exclusion criteria were predefined as follows: presence of active infection or sepsis at admission, chronic inflammatory or autoimmune disease, malignancy, severe hepatic failure, severe renal failure, immunosuppressive therapy, and refusal to participate. Severe renal failure was defined operationally as chronic maintenance renal replacement therapy (dialysis) at the time of admission. Patients with incomplete laboratory data or insufficient serum samples were also excluded from the analysis. A total of 108 patients were screened, of whom 23 were excluded: 13 died before surgery could be performed and therefore did not complete the serial sampling protocol, 8 were receiving chronic dialysis, and 2 had a pathological fracture secondary to malignancy. The remaining 85 patients constituted the study cohort, all of whom underwent surgical treatment. Because patients who died before surgery were not enrolled, this cohort represents patients who survived to operative treatment, and the findings should be interpreted within that scope. The patient selection process and overall study workflow are summarized in Figure 1.

### 2.3. Ethical Approval

The study protocol was approved by the Kütahya Health Sciences University Rectorate, Non-Interventional Clinical Research Ethics Committee (Approval No. 2025/14-54, Date: 16 December 2025), before the enrollment of the first patient. The committee classified the study as a prospective cohort study to be conducted on biological material obtained during routine clinical examination, laboratory testing, and treatment. In accordance with this approval, presepsin was measured exclusively on residual serum remaining after routine clinical laboratory analysis; no blood was drawn from any patient for the sole purpose of this study, and no study-specific intervention or additional procedure was performed. All presepsin assays and all study analyses were carried out after the date of ethics committee approval. Written informed consent for the use of residual biological material and clinical data for research purposes was obtained from all patients or their legally authorized representatives. The study was conducted in accordance with the principles of the Declaration of Helsinki.

### 2.4. Data Collection

Demographic data including age and sex, fracture type, and comorbidities (hypertension, diabetes mellitus, and cardiovascular disease) were obtained from medical records. American Society of Anesthesiologists (ASA) physical status class, the operative procedure performed, the length of hospital stay and, for non-survivors, the hospital day of death were also retrieved from hospital records. Comorbid conditions were recorded individually and were not summarized using composite indices. Causes of death could not be formally adjudicated: all in-hospital deaths were registered as cardiopulmonary arrest, no autopsy was performed in any patient, and no independent adjudication process was available. No post-discharge follow-up was performed.

### 2.5. Blood Sampling and Laboratory Analysis

Routine samples were collected into 5 mL gel-separator biochemistry tubes (13 × 100 mm; Sarstedt AG & Co. KG, Nümbrecht, Germany) as part of standard clinical care. After arrival at the laboratory, the tubes were allowed to stand for 10–20 min and then centrifuged at 3000 rpm for 20 min (NF 1200; Nüve Sanayi Malzemeleri İmalat ve Ticaret A.Ş., Ankara, Türkiye). Residual serum was transferred into secondary microcentrifuge tubes and stored at −20 °C until analysis, with a maximum storage duration of approximately six months and a single freeze–thaw cycle.

Serum presepsin concentrations were measured using a commercially available enzyme-linked immunosorbent assay (ELISA) kit (Human Presepsin ELISA Kit, Cat. No. E3754Hu; Bioassay Technology Laboratory [BT Lab], Shanghai, China) according to the manufacturer’s instructions. All measurements were performed in duplicate, and mean values were used for analysis. The assay was based on the sandwich ELISA principle, with absorbance measured at 450 nm on a microplate reader (800 TS; BioTek Instruments, Inc., Winooski, VT, USA)and concentrations calculated from the standard curve. According to the manufacturer, the kit has a sensitivity of 2.39 ng/L, a measurement range of 5–1000 ng/L, and intra- and inter-assay coefficients of variation below 10%. Presepsin concentrations are reported in ng/L. All laboratory analyses were performed by laboratory personnel who were blinded to the clinical outcomes of the patients. All samples were analysed in a single batch at the end of the study period. Prior to analysis, all samples were visually inspected, and hemolyzed, lipemic, or icteric samples were excluded from the assay.

Routine laboratory parameters, including white blood cell count, lymphocyte count, monocyte count, platelet count, aspartate aminotransferase (AST), alanine aminotransferase (ALT), serum creatinine, and C-reactive protein (CRP), were analyzed in the hospital central laboratory. Biochemical parameters (CRP, creatinine, AST, ALT) were measured on a Cobas c703 analyser (Roche Diagnostics GmbH, Mannheim, Germany), and complete blood counts (white blood cell, lymphocyte, monocyte and platelet counts) on a Sysmex XN-1000 analyser (Sysmex Corporation, Kobe, Japan). The estimated glomerular filtration rate (eGFR) was calculated from serum creatinine on Days 1, 3, and 5 using the 2021 CKD-EPI creatinine equation without a race coefficient. Acute kidney injury was defined according to KDIGO criteria as an increase in serum creatinine of ≥0.3 mg/dL or a ≥1.5-fold rise relative to the Day 1 value.

### 2.6. Statistical Analysis

Statistical analyses were performed using SPSS software (version 25.0; IBM Corp., Armonk, NY, USA) and R (version 4.3). Continuous variables were tested for normality using the Shapiro–Wilk test. Normally distributed data were expressed as mean ± standard deviation, while non-normally distributed data were presented as median (interquartile range). Comparisons between survivors and non-survivors were performed using the Student’s *t*-test or the Mann–Whitney U test, as appropriate. Categorical variables were compared using the chi-square test or, where expected cell counts were below five, an exact test; fracture type was treated as a single categorical variable and assessed in one overall comparison using the Fisher–Freeman–Halton exact test rather than by separate category-wise tests. Temporal changes across Days 1, 3, and 5 and their difference between outcome groups were analysed with a prespecified linear mixed-effects model fitted to log-transformed presepsin concentrations, with a random intercept for each patient and fixed effects for time (categorical), outcome group, and their interaction; the group-by-time interaction is reported as a ratio of geometric means with 95% confidence intervals, together with an overall likelihood-ratio test. As a sensitivity analysis, the same model was refitted as a generalized estimating equation with a gamma distribution, a log link, and an exchangeable working correlation structure.

Receiver operating characteristic (ROC) curve analysis was conducted to evaluate the predictive performance of presepsin, CRP, and platelet counts for in-hospital mortality. The area under the curve (AUC) with 95% confidence intervals was calculated. The optimal cutoff values were determined using the Youden index. Because the same dataset was used to select the time point, to derive the cutoff, and to estimate the regression coefficients, internal validation was performed by bootstrap resampling (2000 replications) to quantify optimism and to characterise the stability of the Youden-derived cutoff. All Day 5 analyses are accordingly reported as exploratory. A *p*-value < 0.05 was considered statistically significant.

### 2.7. Multivariate Logistic Regression Analysis

Multivariable logistic regression analysis was performed to identify predictors of in-hospital mortality. Given the limited number of mortality events (*n* = 17), a formal univariate screening approach followed by multivariable modeling was not pursued, as this could lead to overfitting and unstable estimates. Instead, variables were selected a priori based on clinical relevance and biological plausibility. Day 5 presepsin level was included as the primary biomarker of interest, while age was selected as an established clinical risk factor for hip fracture mortality. The primary model was deliberately restricted to two covariates, in accordance with the commonly recommended events-per-variable principle (approximately 5–10 events per variable). Prespecified sensitivity models additionally included Day 1 eGFR, Day 5 eGFR, or fracture type; because these models exceed the recommended events-per-variable ratio, they are reported as exploratory and are accompanied by bootstrap optimism-corrected estimates of discrimination. The incremental value of Day 5 presepsin was assessed against a baseline clinical model comprising age, ASA class, intertrochanteric fracture type, and Day 5 eGFR, using the likelihood-ratio test, the change in AUC with DeLong testing, the Hosmer–Lemeshow calibration test, and decision-curve analysis. Odds ratios (ORs) with 95% confidence intervals (CIs) were calculated. The predictive performance of biomarkers was compared using DeLong testing. Spearman correlation analysis was performed to assess associations between presepsin and other inflammatory parameters. Serum presepsin was measured at three pre-specified serial time points (Days 1, 3, and 5); all three time points were analysed and are reported in full in the Results, and none was added after data inspection, and none was added after data inspection. Among these pre-specified time points, Day 5 presepsin showed the strongest association with in-hospital mortality and was used as the primary biomarker for the ROC and multivariable analyses; because the time point, the cutoff, and the model were all derived from the same dataset, this finding is interpreted as exploratory and requiring confirmation in independent cohorts. To ensure data integrity, all laboratory values were verified against the source hospital records before the final analysis.

## 3. Results

A total of 85 patients aged 65 years and older with hip fractures were included in the study. Of these, 68 were classified as survivors and 17 as non-survivors based on in-hospital outcomes.

The mean age was 79.3 ± 9.4 years in survivors and 76.0 ± 8.2 years in non-survivors, with no statistically significant difference (*p* = 0.19). Sex distribution was similar between groups. Fracture type, analysed as a single categorical variable in one overall comparison, differed significantly between outcome groups (Fisher–Freeman–Halton exact test, *p* = 0.039): in-hospital mortality was 31.2% among patients with intertrochanteric fractures, compared with 7.4% for cervical fractures and 0% for both basicervical and subtrochanteric fractures. The prevalence of hypertension, diabetes mellitus, and cardiovascular disease did not differ significantly between groups (Table 1).

All 85 patients underwent surgical treatment; no patient was managed non-operatively. Perioperative characteristics and the timing of in-hospital death are summarised in Table 2. The median length of hospital stay was 7 days (IQR 7–9) and did not differ between groups (*p* = 0.51). All 17 deaths occurred between hospital days 5 and 12 (median day 7, IQR 6–9); no patient died before Day 5, so the entire cohort was alive and hospitalised at the Day 5 landmark. Hemiarthroplasty was the predominant procedure, reflecting the institutional policy of arthroplasty for osteoporotic intertrochanteric fractures in order to permit immediate full weight-bearing; in-hospital mortality did not differ significantly between procedures (*p* = 0.35).

Serial renal function data are shown in Table 3. Renal function was not worse in non-survivors; on the contrary, the estimated glomerular filtration rate was significantly higher in non-survivors at all three time points, and acute kidney injury was less frequent in this group. Day 5 presepsin did not correlate with eGFR at any time point (Spearman ρ = 0.12 on Day 1, 0.17 on Day 3, and 0.14 on Day 5; all *p* > 0.11), nor with serum creatinine (ρ = 0.130, *p* = 0.236).

AST and ALT levels remained within normal ranges in both groups, with no significant differences observed at any time point (all *p* > 0.05) (Table 4).

White blood cell counts were significantly higher in non-survivors on Day 1 (*p* = 0.039) and significantly lower on Day 3 (*p* = 0.047), and lymphocyte counts were significantly lower in non-survivors on Day 1 (*p* = 0.004). White blood cell counts on Day 5, lymphocyte counts on Days 3 and 5, and monocyte and platelet counts at all three time points did not differ significantly between groups (Table 5).

CRP levels increased in both groups but did not reach statistical significance at any time point (all *p* > 0.05) (Table 6).

**Figure 2:**
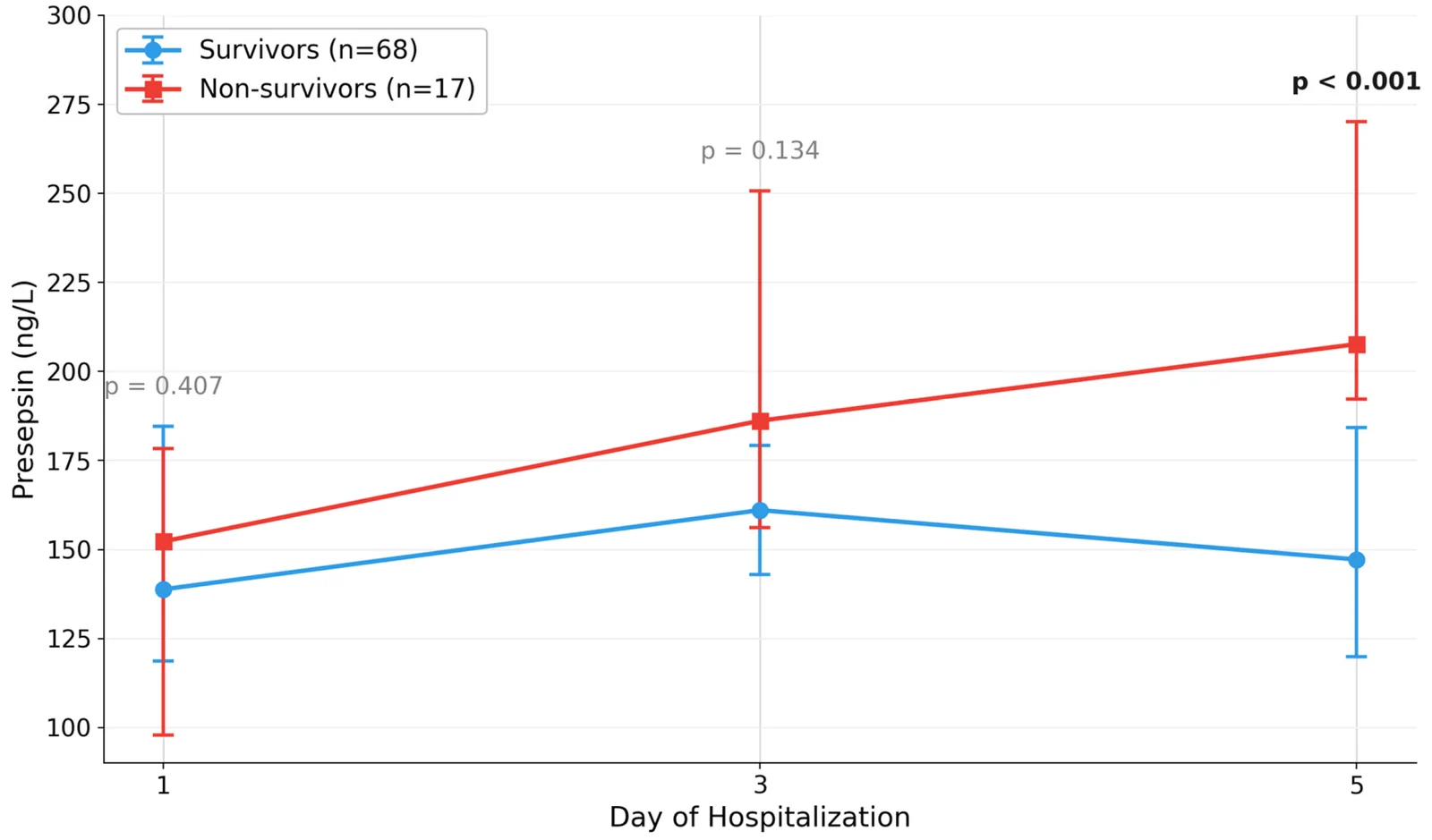
Serial presepsin concentrations in survivors (*n* = 68) and non-survivors (*n* = 17) on days 1, 3, and 5 of hospitalization. Points represent medians and error bars the interquartile range. Between-group comparisons (Mann–Whitney U test) are shown for each time point; presepsin was significantly higher in non-survivors by day 5 (*p* < 0.001). The corresponding group-by-time interaction was estimated with a linear mixed-effects model fitted to log-transformed values (Day 5 × group ratio 1.76, 95% CI 1.38–2.25; *p* < 0.001).

Presepsin concentrations showed a progressive divergence between groups over time. On Day 1, presepsin levels were similar between survivors and non-survivors (median 138.84 vs. 152.28 ng/L; *p* = 0.407). On Day 3, non-survivors had numerically higher levels (median 186.19 vs. 161.08 ng/L; *p* = 0.134). By Day 5, non-survivors demonstrated significantly elevated presepsin concentrations compared with survivors (median 207.70 vs. 147.21 ng/L; *p* < 0.001) (Table 7, Figure 2). In the prespecified linear mixed-effects model fitted to log-transformed presepsin, the group-by-time interaction was significant overall (likelihood-ratio χ^2^ = 19.73, df = 2, *p* < 0.001). Relative to Day 1, the change in presepsin by Day 5 was 1.76-fold greater in non-survivors than in survivors (95% CI 1.38–2.25; *p* < 0.001), whereas the Day 3 interaction did not reach significance (ratio 1.27, 95% CI 0.99–1.62; *p* = 0.060). The generalized estimating equation sensitivity analysis produced a comparable Day 5 estimate (ratio 1.74, 95% CI 1.32–2.30; *p* < 0.001). Geometric mean presepsin concentrations on Days 1, 3, and 5 were 151.4, 165.7, and 144.4 ng/L in survivors and 133.7, 185.5, and 224.4 ng/L in non-survivors.

**Table 7:** Comparison of Presepsin Levels Between Survivors and Non-Survivors.

Time Point	Survivors (*n* = 68)	Non-Survivors (*n* = 17)	*p*-Value
Presepsin Day 1 (ng/L)	138.84 (118.62–184.63)	152.28 (97.83–178.34)	0.407
Presepsin Day 3 (ng/L)	161.08 (142.94–179.25)	186.19 (156.10–250.72)	0.134
Presepsin Day 5 (ng/L)	147.21 (119.96–184.35)	207.70 (192.32–270.10)	<0.001 ***

Data are presented as median (interquartile range); between-group comparisons used the Mann–Whitney U test (non-normal distribution; Shapiro–Wilk test). A linear mixed-effects model fitted to log-transformed presepsin demonstrated a significant group-by-time interaction (Day 5 × group ratio 1.76, 95% CI 1.38–2.25, *p* < 0.001; overall likelihood-ratio χ^2^ = 19.73, df = 2, *p* < 0.001). ng/L, nanograms per liter. *** *p* < 0.001.

Spearman correlation analysis revealed no significant correlations between Day 5 presepsin and other inflammatory parameters, including CRP (r = −0.071, *p* = 0.516), platelet count (r = 0.065, *p* = 0.552), WBC (r = 0.128, *p* = 0.243), and lymphocyte count (r = −0.083, *p* = 0.450) (Table 8). The absence of correlation indicates that presepsin does not simply track conventional inflammatory markers; it does not by itself establish incremental prognostic value, which was therefore evaluated separately against a baseline clinical model, as reported below.

**Table 8:** Spearman Correlation Matrix Between Day 5 Presepsin and Other Inflammatory Parameters.

	Presepsin	CRP	WBC	Lymphocytes	Monocytes	Platelets
Presepsin	1.000	−0.071	0.128	−0.083	0.024	0.065
CRP	−0.071	1.000	0.154	−0.074	0.095	−0.019
WBC	0.128	0.154	1.000	−0.184	0.165	−0.008
Lymphocytes	−0.083	−0.074	−0.184	1.000	0.192	0.173
Monocytes	0.024	0.095	0.165	0.192	1.000	−0.010
Platelets	0.065	−0.019	−0.008	0.173	−0.010	1.000

Values represent Spearman’s rank correlation coefficients (ρ) for Day 5 measurements. None of the correlations between presepsin and the other parameters reached statistical significance: CRP (*p* = 0.516), WBC (*p* = 0.242), lymphocytes (*p* = 0.450), monocytes (*p* = 0.826), and platelets (*p* = 0.552). CRP, C-reactive protein; WBC, white blood cell count.

**Figure 3:**
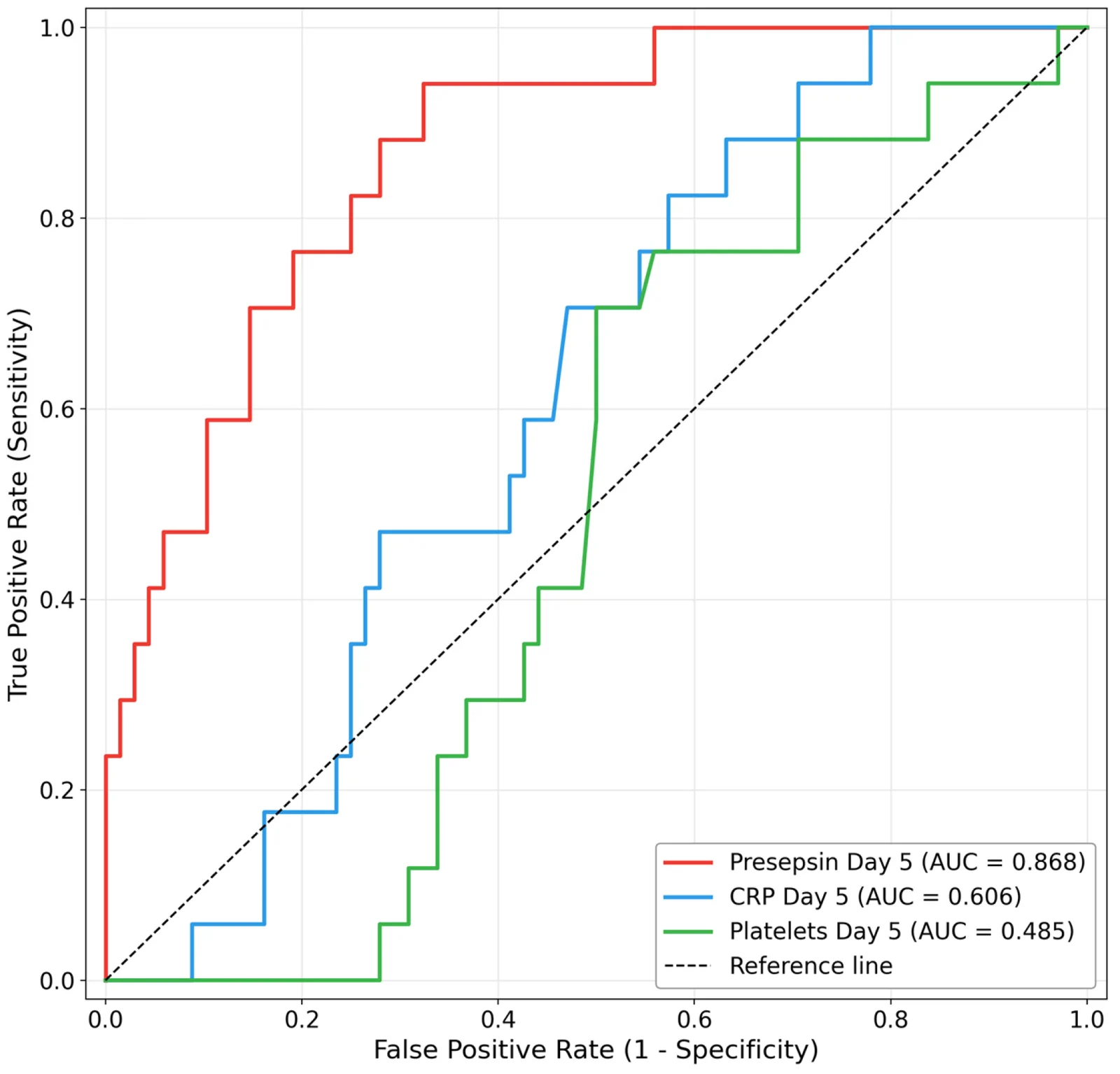
Receiver operating characteristic (ROC) curves for day 5 biomarkers in the prediction of in-hospital mortality. Areas under the curve were 0.868 for presepsin, 0.606 for CRP, and 0.485 for platelet count, corresponding to the values reported in the text and in Table 9. Day 5 presepsin showed significantly greater discriminatory performance than both CRP (DeLong z = 3.023, *p* = 0.003) and platelet count (z = 4.333, *p* < 0.001). The diagonal dashed line represents the reference (no-discrimination) line. AUC, area under the curve; CRP, C-reactive protein.

ROC curve analysis demonstrated that Day 5 presepsin had the highest discriminatory power for predicting in-hospital mortality, with an AUC of 0.868 (95% CI: 0.774–0.945). The optimal cutoff value, determined using the Youden index, was 169.54 ng/L, yielding a sensitivity of 94.1%, specificity of 67.6%, positive predictive value of 42.1%, and negative predictive value of 97.9%. CRP Day 5 showed an AUC of 0.606 (95% CI: 0.473–0.730), and platelet count Day 5 showed an AUC of 0.485 (95% CI: 0.347–0.615). DeLong testing confirmed that the AUC of Day 5 presepsin was significantly superior to both CRP (z = 3.023, *p* = 0.003) and platelet count (z = 4.333, *p* < 0.001) (Table 9, Figure 3). Bootstrap internal validation (2000 replications) indicated negligible optimism for Day 5 presepsin (mean optimism 0.002; optimism-corrected AUC 0.866, bootstrap 95% CI 0.778–0.951). The Youden-derived cutoff, however, was unstable across resamples (median 177.6 ng/L, 95% range 169.5–207.7 ng/L), indicating that the value of 169.54 ng/L should be regarded as cohort-specific and exploratory rather than as a threshold ready for clinical application. The negative predictive value of 97.9% is likewise dependent on the mortality prevalence of this cohort and was obtained in the same sample from which the cutoff was derived.

Multivariate logistic regression analysis, including Day 5 presepsin level and age as a priori selected variables, demonstrated that Day 5 presepsin was significantly associated with in-hospital mortality (OR = 1.029, 95% CI: 1.015–1.044, *p* < 0.001). Age did not retain statistical significance in the model (OR = 0.964, 95% CI: 0.890–1.044, *p* = 0.361). The Hosmer–Lemeshow test indicated adequate model fit (χ^2^ = 9.013, *p* = 0.341) (Table 10). When expressed per clinically meaningful increments, the odds of in-hospital mortality increased by approximately 34% per 10 ng/L (OR = 1.34, 95% CI: 1.16–1.54) and more than fourfold per 50 ng/L (OR = 4.25, 95% CI: 2.08–8.69) increase in Day 5 presepsin; the assumption of linearity for the continuous presepsin term was supported by a non-significant Box–Tidwell interaction (*p* = 0.48). In the prespecified sensitivity models, the association between Day 5 presepsin and mortality was unchanged after adjustment for renal function (with Day 1 eGFR: OR per 10 ng/L 1.40, 95% CI 1.17–1.67; with Day 5 eGFR: OR 1.41, 95% CI 1.17–1.70) and after adjustment for intertrochanteric fracture type (OR 1.33, 95% CI 1.15–1.54). Bootstrap optimism-corrected AUCs were 0.869 for the primary model and 0.883 for the model additionally including Day 5 eGFR. Because these sensitivity models include three covariates for 17 events, they are reported as exploratory.

The incremental value of Day 5 presepsin was assessed against a baseline clinical model comprising age, ASA class, intertrochanteric fracture type, and Day 5 eGFR (Table 11). Adding Day 5 presepsin improved discrimination from an AUC of 0.811 to 0.935 (ΔAUC 0.125; DeLong z = 2.44, *p* = 0.015) and produced a significant improvement in model fit (likelihood-ratio χ^2^ = 23.63, df = 1, *p* < 0.001). Calibration of the extended model was adequate (Hosmer–Lemeshow χ^2^ = 3.26, *p* = 0.354), and decision-curve analysis showed a higher net benefit than both the baseline model and a treat-all strategy across threshold probabilities from 0.05 to 0.50. After bootstrap correction, the optimism-corrected AUCs were 0.776 and 0.906, respectively. These models include up to five covariates for 17 events and are therefore reported as exploratory.

**Table 11:** Incremental Predictive Value of Day 5 Presepsin over a Baseline Clinical Model.

Model	AUC (Apparent)	AUC (Optimism-Corrected)	Likelihood-Ratio test
Baseline clinical model †	0.811	0.776	Reference
Baseline model + Day 5 presepsin	0.935	0.906	χ^2^ = 23.63, *p* < 0.001

† Baseline clinical model: age, ASA class, intertrochanteric fracture type, and Day 5 eGFR. Optimism-corrected AUCs were obtained by bootstrap resampling with 1000 replications. The change in AUC was tested with the DeLong method (ΔAUC 0.125; z = 2.44, *p* = 0.015). Calibration of the extended model was adequate (Hosmer–Lemeshow χ^2^ = 3.26, *p* = 0.354). AUC, area under the receiver operating characteristic curve; eGFR, estimated glomerular filtration rate.

The linear mixed-effects analysis described above demonstrated a significant group-by-time interaction for presepsin, indicating that the temporal trajectories differed between survivors and non-survivors. The corresponding interaction for CRP was not significant (Day 5 × group ratio 1.50, 95% CI 0.83–2.72; *p* = 0.176). Among the other serially measured parameters, platelet counts changed significantly over time in survivors (*p* = 0.037) but not in non-survivors (*p* = 0.191), whereas lymphocyte counts changed significantly in non-survivors (*p* = 0.005) but not in survivors (*p* = 0.113); these within-group comparisons are descriptive and were not used to test the group-by-time interaction.

## 4. Discussion

Hip fractures in older adults remain a major public health problem worldwide, associated with significant morbidity and mortality despite advances in surgical techniques, anesthesia, and perioperative care [1,2]. In-hospital mortality rates range from 5% to 10%, while one-year mortality can reach up to 30% [3,4]. Early identification of patients at high risk of adverse outcomes is therefore critical to improving survival and optimizing the use of healthcare resources [5]. In this prospective study, we evaluated the prognostic role of serial presepsin measurements alongside routine laboratory markers in older adults with hip fractures. Our findings highlight Day 5 presepsin as the strongest predictor of in-hospital mortality, significantly outperforming CRP and platelet counts, while early lymphopenia on Day 1 was associated with mortality in unadjusted comparisons only.

### 4.1. Key Findings and Their Biological Interpretation

The most striking finding of this study was the progressive divergence in presepsin concentrations between survivors and non-survivors over the hospital course. While presepsin levels were similar between groups at admission and Day 3, non-survivors exhibited a significant rise by Day 5 (median 207.70 vs. 147.21 ng/L; *p* < 0.001). The linear mixed-effects model confirmed that the temporal trajectories differed between groups, the change from Day 1 to Day 5 being 1.76-fold greater in non-survivors (95% CI 1.38–2.25; *p* < 0.001). This trajectory suggests that presepsin reflects the cumulative burden and persistence of innate immune activation rather than the acute response to injury alone.

Among conventional markers, lymphocyte counts on Day 1 were significantly lower in non-survivors (0.65 vs. 1.06 × 10^3^/µL; *p* = 0.004). Early lymphopenia has been recognized as a marker of immune suppression and poor prognosis in critically ill and surgical populations [6,7]. In older adults with hip fractures, the neutrophil-to-lymphocyte ratio (NLR) has been shown to predict postoperative mortality [6,7], and our findings support the prognostic relevance of admission lymphocyte count as an early indicator of immune competence in this population.

CRP levels followed a characteristic trajectory in both groups, peaking on Day 3. In descriptive within-group comparisons, CRP changed significantly over time in survivors (*p* < 0.001) but not in non-survivors (*p* = 0.305); these within-group tests are reported for completeness and do not constitute a test of the group-by-time interaction, which was not significant for CRP in the mixed-effects model (Day 5 × group ratio 1.50, 95% CI 0.83–2.72; *p* = 0.176). Between-group differences in CRP did not reach statistical significance at any time point. This is consistent with previous studies demonstrating that CRP, while sensitive to inflammation, lacks specificity for predicting mortality in hip fracture patients [16,17]. Platelet counts and monocyte levels similarly showed no significant between-group differences.

### 4.2. Presepsin and Mechanisms of Mortality Prediction

Presepsin (sCD14-ST) is a soluble fragment of the cluster of differentiation 14 receptor, released during activation of the monocyte–macrophage system in response to infectious and inflammatory stimuli [9,15,18]. While presepsin has been extensively studied in sepsis, where it correlates with disease severity and mortality [19,20], its role in non-infectious inflammatory conditions has received less attention. Notably, Kang et al. demonstrated that presepsin was superior to procalcitonin and CRP for early detection of infection in trauma patients, as it was not elevated by trauma stress alone [15]. Our study extends these findings by demonstrating that presepsin is also a valuable prognostic biomarker in older adults with hip fractures in the absence of overt sepsis. The persistent elevation of presepsin in non-survivors likely reflects sustained monocyte–macrophage activation driven by trauma, surgical stress, tissue ischemia, and possibly subclinical infectious complications.

Spearman correlation analysis revealed no significant associations between Day 5 presepsin and other inflammatory parameters (CRP, WBC, platelets, lymphocytes), which suggests that presepsin may capture an aspect of immune activation not reflected by conventional markers. The absence of correlation does not, however, demonstrate incremental prognostic value; that question was addressed separately by formal comparison against a baseline clinical model, in which the addition of Day 5 presepsin improved discrimination (ΔAUC 0.125; *p* = 0.015) and model fit (likelihood-ratio χ^2^ = 23.63, *p* < 0.001), albeit in exploratory analyses that require external validation.

### 4.3. Roc Analysis and Discriminatory Performance

ROC curve analysis confirmed Day 5 presepsin as the strongest predictor of in-hospital mortality, with an AUC of 0.868 (95% CI: 0.774–0.945), which remained essentially unchanged after bootstrap optimism correction (0.866). DeLong testing demonstrated that this was significantly superior to CRP (AUC = 0.606; *p* = 0.003) and platelet counts (AUC = 0.485; *p* < 0.001). The Youden-derived cutoff of 169.54 ng/L yielded high sensitivity (94.1%) and a high negative predictive value (97.9%). These operating characteristics must, however, be interpreted with caution: the cutoff varied between 169.5 and 207.7 ng/L across bootstrap resamples, and the negative predictive value depends on the mortality prevalence of this cohort and was obtained in the same sample from which the threshold was derived. No clinical decision should therefore be based on this threshold without external validation.

Our AUC value of 0.868 for presepsin compares favorably with previously reported AUC values for other prognostic tools in hip fracture patients. A recent systematic review by Badosa-Collell et al. [5] reported AUC values of 0.82 for the ASAgeCoGeCC and Almelo scores for 30-day mortality prediction. The HALP score, combining hemoglobin, albumin, lymphocyte, and platelet values, showed good predictive performance for 90-day mortality in older adults with hip fractures [21].

### 4.4. Comparison with Routine Biomarkers

Although CRP is widely used as an inflammatory marker in hip fracture patients, its specificity for predicting mortality is limited [16]. Kim et al. demonstrated that elevated preoperative CRP (>10.0 mg/dL) was an independent predictor of one-year mortality [17], but its performance in predicting in-hospital mortality was less robust. In our study, CRP failed to significantly differentiate survivors from non-survivors at any time point. Niessen et al. similarly reported that CRP at admission did not predict in-hospital mortality in older adults with hip fractures [16]. These findings suggest that CRP reflects the magnitude of acute inflammation but may not adequately capture the immune dysregulation that drives early mortality.

The absence of significant prognostic contributions from AST, ALT, platelet counts, and monocyte counts further highlights the need for biomarkers with greater specificity for the pathological processes driving mortality in this population.

### 4.5. Relation to Fracture Type and Clinical Features

When fracture type was analysed as a single categorical variable in one overall comparison, it differed significantly between outcome groups (Fisher–Freeman–Halton exact test, *p* = 0.039). Mortality was concentrated among patients with intertrochanteric fractures (31.2%), compared with 7.4% for cervical fractures and none of the patients with basicervical or subtrochanteric fractures. This pattern is consistent with the greater blood loss, longer operative time, and frailer patient profile typically associated with trochanteric fracture patterns. Treatment strategies and outcomes are known to vary across hip-fracture patterns and across the available fixation and arthroplasty options [22]. In our institution, all intertrochanteric fractures were treated with hemiarthroplasty, a deliberate institutional policy intended to permit immediate full weight-bearing in patients with severely osteoporotic bone; as a consequence, fracture pattern and surgical procedure were almost completely confounded in this cohort, and their separate contributions to mortality cannot be disentangled. Comorbidities such as hypertension, diabetes, and cardiovascular disease did not differ significantly between groups, and neither age nor ASA class explained the mortality difference. These observations are consistent with prior literature demonstrating that pre-existing comorbidity indices often fail to adequately predict in-hospital mortality in older adults with hip fractures [3,4].

The in-hospital mortality of 20% observed in this cohort is higher than the 5–10% commonly reported, and its interpretation requires attention to how the cohort was assembled. Of 108 screened patients, 13 died before surgery could be performed and were therefore not enrolled; among all screened patients, in-hospital mortality was consequently at least 27.8%. The figure of 20% therefore describes a cohort from which the earliest and most severely compromised deaths had already been removed, rather than an inflated estimate. The predominance of intertrochanteric fractures (56.5% of the cohort, with a mortality of 31.2%) and of ASA class III–IV patients (96.5%) further contributes to the observed rate. These features should be borne in mind when comparing our results with those of unselected hip-fracture series.

### 4.6. Clinical Implications

From a clinical perspective, serial presepsin measurement may have value for perioperative risk stratification in older adults with hip fractures, but the present data do not support any specific clinical action. The Day 5 cutoff derived here was unstable across bootstrap resamples and was obtained in the same cohort in which it was evaluated; the high negative predictive value is likewise dependent on the mortality prevalence of this cohort and cannot be transferred to populations with a different baseline risk. Accordingly, we do not propose that Day 5 presepsin be used to guide early mobilization, discharge planning, intensive care admission, infection surveillance, or any modification of treatment protocols.

It should also be noted that a Day 5 biomarker, whatever its prognostic value, cannot inform initial triage decisions. Future studies should explore multimarker risk models incorporating both early and late biomarkers alongside clinical variables such as frailty, renal function, and surgical delay, with prospective external validation.

### 4.7. Strengths and Limitations

This study has several strengths, including its prospective design, serial biomarker measurements at predefined time points (Days 1, 3, and 5), complete serial renal function data, the use of a longitudinal mixed-effects model with an explicitly estimated group-by-time interaction, bootstrap internal validation, and direct statistical comparison of novel and conventional markers using DeLong testing.

Limitations must be acknowledged. First, this was a single-center study with a relatively small sample size (85 patients, 17 mortality events), which limits generalizability and statistical power. Second, only in-hospital mortality was assessed; long-term outcomes (30-day, 6-month, or 1-year mortality) were not evaluated. Third, although renal function, ASA class, and fracture type were incorporated into sensitivity analyses, other important confounders including frailty, cognitive status, nutritional status, surgical delay, perioperative complications, and intensive care admission could not be assessed. Fourth, the sensitivity models exceed the recommended events-per-variable ratio and are therefore exploratory; residual confounding cannot be excluded. Fifth, the cutoff derived from ROC analysis is specific to the study population, proved unstable on bootstrap resampling, and requires external validation before any clinical implementation. Sixth, comparison with other novel biomarkers such as procalcitonin, IL-6, or D-dimer was not performed. Seventh, patients receiving chronic dialysis were excluded, so the findings cannot be extrapolated to patients with end-stage renal disease. Finally, because Day 5 presepsin can be measured only in patients who survive to Day 5, its prognostic value should be interpreted as reflecting ongoing clinical deterioration rather than as a tool for admission-based triage; in this cohort no patient died before Day 5, so the Day 5 landmark population comprised the entire sample and the analysis was not affected by missing-data survivor bias. Nevertheless, higher Day 5 presepsin concentrations were associated with a shorter interval to death (Spearman ρ = −0.58, *p* = 0.015), so a contribution from terminal deterioration cannot be excluded; discrimination was, however, retained when the landmark was moved to Day 7 (AUC 0.825).

Furthermore, detailed clinical data regarding time to surgery, type of anesthesia, postoperative complications, hospital-acquired infections, intensive care unit admission, nutritional status, and baseline functional status could not be reliably retrieved from the hospital record system and are therefore not reported. Causes of death could not be adjudicated: all deaths were registered as cardiopulmonary arrest, a documented diagnosis of pulmonary embolism was present in only 2 patients, and autopsy was not performed. These factors are known to influence both mortality and systemic inflammatory responses in older adults with hip fractures, and their absence represents an important limitation. Notably, conventional infection markers did not differ between groups on Day 5 (CRP *p* = 0.178; WBC *p* = 0.117), and renal function was better rather than worse in non-survivors, so the observed presepsin elevation is unlikely to be explained by overt infection or by declining glomerular filtration alone; nonetheless, the possibility of residual confounding cannot be excluded. The elevated presepsin concentrations observed in non-survivors should therefore be interpreted as an association rather than as evidence of an independent causal relationship. Consequently, the present findings should be regarded as exploratory and hypothesis-generating, requiring confirmation in larger, well-characterized multicenter cohorts that incorporate these clinical variables.

### 4.8. Future Directions

Future research should focus on validating the prognostic value of Day 5 presepsin in larger, multicenter cohorts and on external validation of the threshold identified here. Although the present analysis adjusted for renal function and found the association to be essentially unchanged, confirmation in cohorts spanning a wider range of glomerular filtration is required [23,24], since patients receiving chronic dialysis were excluded. Prospective studies could evaluate whether presepsin-guided monitoring translates into improved clinical outcomes. Comparative studies with procalcitonin, IL-6, and D-dimer could clarify presepsin’s relative position in prognostic hierarchies for orthopedic trauma patients.

## 5. Conclusions

Serial presepsin measurement identifies a progressive divergence in innate immune activation between survivors and non-survivors among older adults with hip fractures. Day 5 presepsin demonstrated the strongest predictive performance for in-hospital mortality (AUC = 0.868; optimism-corrected 0.866), significantly outperforming CRP and platelet counts by DeLong testing, and the association persisted after adjustment for age, ASA class, fracture type, and estimated glomerular filtration rate. These findings nevertheless represent an exploratory association derived from a single-centre cohort in which the time point, the threshold, and the model were selected from the same data. External validation in larger, multicentre cohorts is required before serial presepsin measurement can be considered for use in perioperative risk stratification.

## Figures and Tables

**Table 1:** Demographic and Clinical Characteristics of Survivors and Non-Survivors.

Variable	Survivors (*n* = 68)	Non-Survivors (*n* = 17)	*p*-Value
Age (years, mean ± SD)	79.3 ± 9.4	76.0 ± 8.2	0.19
Male sex, *n* (%)	26 (38.2%)	8 (47.1%)	0.58
Female sex, *n* (%)	42 (61.8%)	9 (52.9%)	—
Cervical fracture, *n* (%)	25 (36.8%)	2 (11.8%)	0.039 * †
Basicervical fracture, *n* (%)	4 (5.9%)	0 (0%)	—
Intertrochanteric fracture, *n* (%)	33 (48.5%)	15 (88.2%)	—
Subtrochanteric fracture, *n* (%)	6 (8.8%)	0 (0%)	—
Hypertension, *n* (%)	34 (50.0%)	9 (52.9%)	0.83
Diabetes mellitus, *n* (%)	21 (30.9%)	6 (35.3%)	0.72
Cardiovascular disease, *n* (%)	23 (33.8%)	7 (41.2%)	0.57

Continuous variables are presented as mean ± standard deviation and compared using the Student’s *t*-test; categorical variables are presented as *n* (%) and compared using the chi-square or Fisher’s exact test. SD, standard deviation. * *p* < 0.05 was considered statistically significant. † Fracture type was compared as a single categorical variable across all four categories in one overall Fisher–Freeman–Halton exact test (*p* = 0.039); category-specific *p*-values are therefore not reported.

**Table 2:** Perioperative Characteristics and Timing of In-Hospital Death.

Variable	Survivors (*n* = 68)	Non-Survivors (*n* = 17)	*p*-Value
Surgical treatment, *n* (%)	68 (100%)	17 (100%)	—
ASA class II, *n* (%)	2 (2.9%)	1 (5.9%)	0.82 †
ASA class III, *n* (%)	59 (86.8%)	14 (82.4%)	—
ASA class IV, *n* (%)	7 (10.3%)	2 (11.8%)	—
Hemiarthroplasty, *n* (%)	58 (85.3%)	17 (100%)	0.35 †
Proximal femoral nail (PFNA), *n* (%)	6 (8.8%)	0 (0%)	—
Total hip arthroplasty, *n* (%)	4 (5.9%)	0 (0%)	—
Length of hospital stay (days)	7 (7–8)	7 (6–9)	0.51
Hospital day of death, median (IQR)	—	7 (6–9)	—
Death on hospital days 5–6, *n* (%)	—	6 (35.3%)	—
Death on hospital day ≥ 7, *n* (%)	—	11 (64.7%)	—

Categorical variables are presented as *n* (%); continuous variables as median (interquartile range). ASA, American Society of Anesthesiologists physical status classification; IQR, interquartile range; PFNA, proximal femoral nail antirotation. † ASA class and operative procedure were each compared as a single categorical variable in one overall exact test; category-specific *p*-values are therefore not reported.

**Table 3:** Renal Function in Survivors and Non-Survivors.

Variable	Survivors (*n* = 68)	Non-Survivors (*n* = 17)	*p*-Value
Creatinine Day 1 (mg/dL)	1.04 (0.89–1.31)	0.97 (0.74–1.07)	0.120
Creatinine Day 3 (mg/dL)	1.15 (0.96–1.42)	0.92 (0.81–1.22)	0.042 *
Creatinine Day 5 (mg/dL)	1.19 (1.08–1.46)	1.01 (0.79–1.25)	0.033 *
eGFR Day 1 (mL/min/1.73 m^2^)	59.8 (50.4–71.9)	82.4 (57.7–87.2)	0.034 *
eGFR Day 3 (mL/min/1.73 m^2^)	52.5 (43.1–67.7)	71.5 (55.5–86.4)	0.016 *
eGFR Day 5 (mL/min/1.73 m^2^)	50.9 (40.7–61.0)	60.6 (51.4–87.0)	0.015 *
Acute kidney injury (KDIGO), *n* (%)	15 (22.1%)	1 (5.9%)	0.175

Data are presented as median (interquartile range) and compared using the Mann–Whitney U test, or as *n* (%) compared using Fisher’s exact test. The estimated glomerular filtration rate (eGFR) was calculated with the 2021 CKD-EPI creatinine equation without a race coefficient. Acute kidney injury was defined by KDIGO criteria as an increase in serum creatinine of ≥0.3 mg/dL or a ≥1.5-fold rise from the Day 1 value. KDIGO, Kidney Disease: Improving Global Outcomes. * *p* < 0.05.

**Table 4:** Comparison of Liver Enzyme Levels Between Survivors and Non-Survivors.

Parameter	Survivors (*n* = 68)	Non-Survivors (*n* = 17)	*p*-Value
AST Day 1 (U/L)	37.87 ± 19.07	28.92 ± 14.24	0.074
AST Day 3 (U/L)	37.38 (20.55–51.56)	32.07 (24.79–38.77)	0.262
AST Day 5 (U/L)	37.07 (20.06–65.68)	30.00 (20.00–69.59)	0.758
ALT Day 1 (U/L)	14.62 (8.93–22.23)	12.79 (6.66–18.21)	0.342
ALT Day 3 (U/L)	13.62 (8.78–23.40)	20.54 (10.24–30.88)	0.258
ALT Day 5 (U/L)	27.72 (11.48–53.04)	25.00 (19.00–40.00)	0.878

Data are presented as mean ± standard deviation for normally distributed variables (Student’s *t*-test) or as median (interquartile range) for non-normally distributed variables (Mann–Whitney U test). Normality was assessed using the Shapiro–Wilk test. AST, aspartate aminotransferase; ALT, alanine aminotransferase; U/L, units per liter. A *p*-value < 0.05 was considered statistically significant.

**Table 5:** Comparison of Hematological Parameters Between Survivors and Non-Survivors.

Parameter	Survivors (*n* = 68)	Non-Survivors (*n* = 17)	*p*-Value
WBC Day 1 (×10^3^/µL)	10.43 (8.05–14.02)	12.81 (11.09–15.91)	0.039 *
WBC Day 3 (×10^3^/µL)	10.82 ± 3.16	9.18 ± 2.12	0.047 *
WBC Day 5 (×10^3^/µL)	10.32 (8.72–11.45)	10.66 (10.00–13.06)	0.117
Lymphocytes Day 1 (×10^3^/µL)	1.06 ± 0.54	0.65 ± 0.33	0.004 **
Lymphocytes Day 3 (×10^3^/µL)	1.32 (0.96–1.43)	1.21 (0.90–1.64)	0.633
Lymphocytes Day 5 (×10^3^/µL)	1.30 (0.86–1.50)	0.90 (0.60–1.50)	0.435
Monocytes Day 1 (×10^3^/µL)	0.74 (0.59–0.99)	0.69 (0.57–0.81)	0.260
Monocytes Day 3 (×10^3^/µL)	0.74 (0.57–0.97)	0.53 (0.44–0.90)	0.086
Monocytes Day 5 (×10^3^/µL)	0.74 (0.59–0.99)	0.89 (0.62–1.04)	0.415
Platelets Day 1 (×10^3^/µL)	219 (189–270)	207 (175–259)	0.621
Platelets Day 3 (×10^3^/µL)	248.66 ± 70.98	254.29 ± 69.99	0.770
Platelets Day 5 (×10^3^/µL)	248 (209–354)	250 (239–283)	0.856

Data are presented as mean ± standard deviation (Student’s *t*-test) or median (interquartile range) (Mann–Whitney U test), based on the Shapiro–Wilk normality test. WBC, white blood cell count. * *p* < 0.05; ** *p* < 0.01.

**Table 6:** Comparison of C-Reactive Protein Levels Between Survivors and Non-Survivors.

Time Point	Survivors (*n* = 68)	Non-Survivors (*n* = 17)	*p*-Value
CRP Day 1 (mg/L)	107.16 (61.01–153.81)	93.60 (59.55–163.26)	0.886
CRP Day 3 (mg/L)	225.34 ± 81.95	220.62 ± 111.57	0.844
CRP Day 5 (mg/L)	146.62 (95.84–210.93)	167.00 (135.96–218.17)	0.178

Data are presented as mean ± standard deviation (Student’s *t*-test) or median (interquartile range) (Mann–Whitney U test). Complete Day 5 data were available for all 17 non-survivors at every time point. CRP, C-reactive protein; mg/L, milligrams per liter. A *p*-value < 0.05 was considered statistically significant.

**Table 9:** ROC Curve Analysis for the Prediction of In-Hospital Mortality (Day 5 Biomarkers).

Biomarker	AUC (95% CI)	Cutoff	Sensitivity	Specificity	PPV	NPV
Presepsin Day 5	0.868 (0.774–0.945)	169.54 ng/L	94.1%	67.6%	42.1%	97.9%
CRP Day 5	0.606 (0.473–0.730)	121.00 mg/L	82.4%	42.6%	26.4%	90.6%
Platelets Day 5	0.485 (0.347–0.615)	247 ×10^3^/µL	70.6%	50.0%	26.1%	87.2%

Optimal cutoff values were determined using the Youden index. Confidence intervals for the AUC were calculated using bootstrap resampling (2000 iterations). All ROC analyses were performed on complete cases (*n* = 85). DeLong test: Presepsin Day 5 vs. CRP Day 5, z = 3.023, *p* = 0.003; Presepsin Day 5 vs. Platelets Day 5, z = 4.333, *p* < 0.001. AUC, area under the curve; CI, confidence interval; PPV, positive predictive value; NPV, negative predictive value.

**Table 10:** Multivariate Logistic Regression Analysis for In-Hospital Mortality.

Variable	Odds Ratio (OR)	95% Confidence Interval	*p*-Value
Age (years)	0.964	0.890–1.044	0.361
Day 5 Presepsin (ng/L)	1.029	1.015–1.044	<0.001 *

Variables were selected a priori based on clinical relevance. The odds ratio for presepsin represents the change in odds of in-hospital mortality per 1 ng/L increase. For clinically meaningful increments, the OR was 1.34 (95% CI: 1.16–1.54) per 10 ng/L and 4.25 (95% CI: 2.08–8.69) per 50 ng/L; linearity of the continuous presepsin term was confirmed by a non-significant Box–Tidwell interaction (*p* = 0.48). Model calibration was adequate (Hosmer–Lemeshow test: χ^2^ = 9.013, *p* = 0.341). OR, odds ratio; CI, confidence interval. * *p* < 0.05.

## Data Availability

The datasets generated and/or analyzed during the current study are available from the corresponding author upon reasonable request.
